# Impact of *Clostridioides difficile* Therapy on Nosocomial Acquisition of Vancomycin-Resistant Enterococci

**DOI:** 10.3390/ph14111066

**Published:** 2021-10-21

**Authors:** Carlos L. Correa-Martínez, Niklas C. J. Hagemeier, Neele J. Froböse, Stefanie Kampmeier

**Affiliations:** 1 Institute of Hygiene, University Hospital Münster, Robert-Koch-Straße 41, 48149 Münster, Germany; Carlos.Correa@ukmuenster.de (C.L.C.-M.); Niklascarljosef.Hagemeier@ukmuenster.de (N.C.J.H.); 2 Institute of Medical Microbiology, University Hospital Münster, Domagkstraße 10, 48149 Münster, Germany; Neelejudith.Froboese@ukmuenster.de

**Keywords:** CDI, VRE, antimicrobial stewardship, whole genome sequencing

## Abstract

Vancomycin is frequently used for the treatment of *C. difficile* infections (CDI). There are concerns that this might increase the risk of selecting vancomycin resistant enterococci (VRE). Here, we evaluated whether there is an increased risk of VRE acquisition following vancomycin for CDI specific treatment. Patients with CDI, metronidazole, or oral vancomycin treatment and without preexisting VRE were monitored for VRE acquisition. VRE isolates from patients with acquired and preexisting colonization were collected and subjected to whole genome sequencing. In total, 281 patients (median age 56 years, 54% of the male sex) presented with toxin positive *C. difficile*. Of them, 170 patients met the inclusion criteria, comprising 37 patients treated with metronidazole and 133 treated with oral vancomycin. In total, 14 patients meeting the inclusion criteria acquired VRE (vancomycin: *n* = 11; metronidazole: *n* = 3). Statistical analysis revealed no significant differences between both VRE acquisition rates. Genetic comparison of detected VRE isolates resulted in eight clusters of closely related genotypes comprising acquired and preexisting strains. Our results suggest that vancomycin and metronidazole likewise increase the risk of VRE acquisition. Genetic comparison indicates that VRE acquisition is a result of both antibiotic selection and pathogen transmission.

## 1. Introduction

In past decades, *Clostridioides difficile* infections (CDI) have evolved into one of the most common healthcare-associated infections worldwide. In Germany, where surveillance of these infections is mandatory, an incidence of 0.47 cases/1000 patient days could be observed in 2020, of which 11% were classified as severe cases following the criteria of the German infection protection law [1,2].

According to common guidelines, therapeutic management of *C. difficile* infections depends on the severity of symptoms and the number of previous episodes. While formerly metronidazole has been considered the first-line agent treatment of non-severe CDI, since 2021, fidaxomicin and alternately vancomycin are assessed as superior for these indications in the current clinical practice guidelines from the Infectious Diseases Society of America and the Society for Healthcare Epidemiology of America [3]. However, metronidazole was preferred for mild or moderate infections previously but is now considered for these indications in the latest guideline release of the European Society of Clinical Microbiology and Infectious Diseases (ESCMID) [4]. Hence, in the University Hospital Münster (UHM), non-severe CDI infections are treated with metronidazole, while patients with severe CDI infections receive oral vancomycin. 

While the choice of antibiotic agents for different clinical presentations is clearly supported by recent studies [5,6,7], its implications need to be further evaluated. Here, alongside economic health aspects, topics of infection prevention, such as the acquisition of multidrug resistant pathogens, need to be considered. Effects of antibiotics on nosocomial epidemiology of vancomycin-resistant enterococci (VRE) are thereby controversially discussed. There is evidence that the intravenous use of vancomycin is a major risk factor for VRE colonization [8,9] and has been associated with long-term VRE shedding [10]. On the other hand, in the absence of vancomycin therapy, VRE acquisition rates were shown to be ca. 1% at baseline but increased to over 6% after ertapenem application [11]. Additionally, oral vancomycin for *C. difficile* treatment did not result in enhanced VRE carriage rates compared to metronidazole treatment in a multicenter retrospective cohort study conducted in the USA [12]. 

VRE is an emerging pathogen posing a major threat to healthcare systems. Infections with VRE lead to a significantly higher mortality than infections with vancomycin susceptible enterococci [13,14]. Several risk factors for healthcare-associated acquisition of VRE are already characterized [15,16]. Alongside comorbidities, therapeutic interventions, and suboptimal implementation of hygiene measures, environmental persistence and subsequent nosocomial acquisition can be favored by pathogen-associated factors [17,18]. It is conceivable that VRE with specific genetic characteristics are preferably selected in CDI patients receiving antibiotic agents. To examine this hypothesis, we compared VRE colonization rates between previously VRE-negative patients receiving either metronidazole or oral vancomycin as a CDI-specific treatment. VRE isolates of patients with preexisting VRE colonization were compared with those of patients colonized after CDI-specific treatment.

## 2. Results

**Figure 1:**
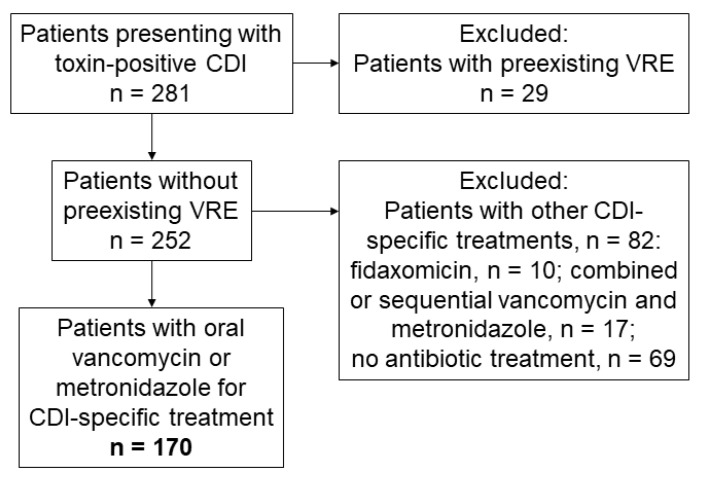
CDI patients meeting inclusion criteria.

During 2018 and 2020, 281 patients (median age 56 years, 54% of male sex) presented with toxin positive CDI, of which 31 were classified as severe cases and 175 as hospital-acquired cases due to the above-mentioned criteria. Of all CDI patients, 145 received oral vancomycin and 40 received metronidazole for CDI-specific treatment. In total, 111 patients did not fulfill the inclusion criteria and had to be excluded due to CDI treatments being different to metronidazole or oral vancomycin mono-treatment (*n* = 82) or preexisting VRE colonization (*n* = 29) (Figure 1).

### 2.1. Characteristics of Included Patients and Onset of VRE

In total, 170 patients (median age 53 years, 55% of male sex) met the inclusion criteria and were further observed. Clinical characteristics potentially favoring VRE can be gathered from Table 1. Of all included patients, 133 received oral vancomycin treatment (4 × 250 mg/day), while in 37 patients metronidazole (3 × 500 mg/day) was the preferred antibiotic agent. In total, 14 patients (metronidazole: 3; vancomycin: 11) acquired VRE after the first application of CDI-specific treatment. Statistical analysis did not result in significant differences between VRE acquisition rates of patients that received treatment with metronidazole and those treated with oral vancomycin (*p* = 0.98). VRE acquisition occurred approximately 21.5 days (metronidazole: 14 days; vancomycin: 25 days) after CDI-specific therapy. 

### 2.2. VRE Genotypes and Genetic Distribution of Strains

**Figure 2:**
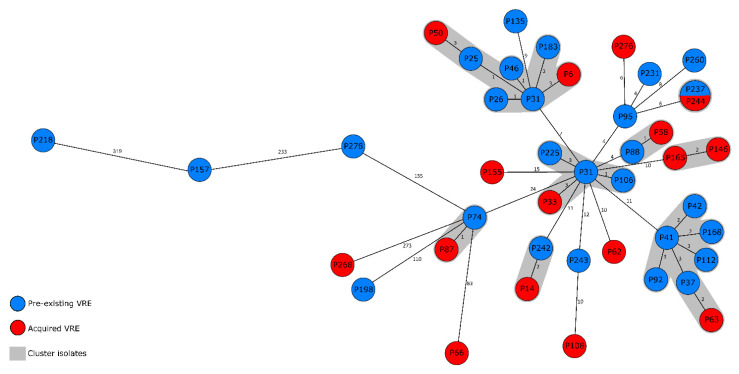
Minimum spanning tree of 43 preexisting (*n* = 27, blue) and acquired (*n* = 16, red) VRE strains based on 1423 target genes pairwise, ignoring missing values and numbered consecutively. Each circle represents one genotype. Connecting lines between genotypes indicate the number of allelic differences. Grey shadings illustrate close genetic relations (≤3 alleles difference between directly connected genotypes).

Of 45 VRE isolates, 43 (16 acquired and 27 preexisting) isolates were available for WGS-based typing. Out of these, 40 (93%) harbored *vanB* and 3 (7%) harbored *vanA.* Among all samples, prevalent MLST STs were ST117 (39 isolates, 91%), ST262 (2 isolates, 5%), ST192, and ST721 (1 isolate each, 2%). CgMLST-based typing resulted in eight clusters of genetically closely-related genotypes comprising 2–8 genotypes (Figure 2, grey highlights), indicating possible intra-hospital VRE transmissions. However, there was no specific clustering of isolates associated with acquired or preexisting VRE (Figure 2).

## 3. Discussion

VRE are an emerging issue to public health worldwide and were therefore classified as microorganisms of high-level priority by the World Health Organization in 2017 [19]. As hospital-acquired VRE are a result of interactions between host-associated factors, such as antibiotic selection and intra-hospital transmission [20,21] we assessed (i) whether oral vancomycin facilitates the acquisition of VRE compared to metronidazole treatment and (ii) if there are pathogen-specific genetic differences favoring VRE acquisition in CDI patients. 

In our study, oral vancomycin did not pose a predisposition for VRE acquisition compared to metronidazole. These results correspond with previous studies. Possible sources of bias in these studies are the analysis of VRE acquisition during CDI prophylaxis with vancomycin [22] and the gender distribution of a patient cohort with more than 90% of male individuals [12,23]. Nevertheless, available data surprisingly do not suggest any association between vancomycin administration and hospital-acquired VRE in CDI affected patients, whereby studies in non-CDI-patients have verified this association [23,24]. This might be due to the significantly lower prevalence of VRE in non-CDI patients, which was <1% in our study period and connotes that CDI-patients are at special risk to acquire VRE.

Moreover, our cgMLST analysis of VRE strains revealed no specific relatedness or clearly distinguished pattern acquired from preexisting VRE isolates. This renders VRE genetic factors unlikely to favor VRE acquisition under CDI treatment. Hence, a more complex interplay of risk factors for this phenomenon needs to be considered, including transmissions via patient surroundings [25] and host gut microbiome changes due to additional antimicrobial treatments [20] facilitating VRE colonization. This context, e.g., the application of cephalosporines, allows VRE selection due to possible disturbance of the normal gut flora [26] and emphasizes the importance of antimicrobial stewardship (AMS) approaches. Our cgMLST analysis did additionally uncover close genetic relations and thereby the possibility of intra-hospital transmissions among isolates originating from patients with preexisting and acquired colonization, supporting the necessity of AMS teams working hand-in=hand with classical infection control specialists for preventing VRE hospital spread. Of note, we mainly determined *vanB*-positive strains comprising ST117, an observation that comes along with current trends in Germany [27] and raises the question whether VRE of this genetic composition do generally have a selective advantage in the hospital setting. Further comparative studies will be needed to address this aspect.

Our study has limitations. As we performed a retrospective observational study, this comes along with common constraints compared with randomized controlled studies. Moreover, we cannot assure that acquired VRE has no association with patient–patient or surface–patient transmission. Prior studies have shown that contact with a confirmed VRE patient is a major risk factor for VRE acquisition [28]. Therefore, we have to rely on the existing VRE control bundle to reduce transmission within the hospital as best as possible. Furthermore, detection of phenotypic vancomycin resistant isolates in the described matter is a limitation, since there was the necessity to select only a few isolated colonies [29]. Nevertheless, up till now, this describes the standard antimicrobial susceptibility testing, thereby offering the possibility of comparisons.

## 4. Materials and Methods

### 4.1. Setting and Study Design

The UHM is a 1427-bed tertiary care center with 57,132 patient admissions in 2019 [30]. In this setting, we conducted a 2-year retrospective cohort study from 2018 to 2020. Patients with episodes of toxin positive CDI receiving metronidazole or oral vancomycin for CDI-specific treatment were included. VRE status at the beginning of CDI treatment was compiled in order to differentiate between patients with and without preexisting VRE colonization. Patients’ characteristics, including demographic data (age, sex) and risk factors for VRE acquisition, such as underlying immunosuppressive diseases (e.g., malignancies, HIV-infection, liver transplantation) and treatments (e.g., long-term dialysis, systemic steroid therapy), were monitored. CDI cases were classified as severe if patients had to be admitted from an ambulatory setting, were transferred to an intensive care unit, underwent colorectal surgery, or died due to CDI infection [2]. CDI was assumed to be hospital-acquired if detected 48 h post-admission. 

### 4.2. Infection Control Measures

In the case of VRE (colonization/infection) or toxin-positive CDI detection, extended hygiene measures were implemented, including contact isolation of patients in separate rooms and extra sanitary facilities. All staff members were advised to wear personal protective equipment, comprising gowns and gloves. Surface disinfection was performed at least once a day. In case of CDI, disinfection was performed with sporicidal disinfectants. Patient with VRE colonization were de-isolated if three anorectal swabs taken in consecutive weeks and without application of antibiotic therapy were negative for VRE. In the case of toxin-positive CDI, isolation was discontinued for patients free of CDI-associated symptoms for at least 48 h. 

### 4.3. CDI Diagnostic Procedure

Unformed stool samples were analyzed in a two-step process for toxins producing *C. difficile* following the current ESCMID standards [4]. In brief, first glutamate dehydrogenase (GDH) was detected using the VIDAS^®^ *C. difficile* GDH system (bioMérieux, Nürtingen, Germany). If GDH positive, *C. difficile* toxin was detected using the Xpert^®^ *C. difficile* BT system (Cepheid^®^, Krefeld, Germany) for confirmation.

### 4.4. VRE Screening, Culture, and Antimicrobial Susceptibility Testing

VRE screening was performed obtaining rectal swabs (5 cm *ab ano*) (Transwab^®^ m40 compliant, mwe, Corsham, Wiltshire, UK), which were streaked to chromogenic selective agar (VRESelect^TM^, Biorad, München, Germany) and incubated for 48 h at 36 °C. Suspected VRE colonies were verified on a species level using MALDI-TOF MS (Bruker Corporation, Bremen, Germany). Antibiotic susceptibility was evaluated in accordance with the current recommendations of the European Committee on Antimicrobial Susceptibility Testing (EUCAST) [31] using the VITEK^®^ 2 system and the minimal inhibition concentration (bioMérieux, Nürtingen, Germany). Confirmation of species identification and glycopeptide resistance (*vanA*, *vanB*, *vanC1*, and *vanC2/3*) was done using the GenoType *Enterococcus*^®^ line probe (Hain Lifescience, Nehren, Germany). 

### 4.5. Whole Genome Sequencing

VRE isolates from CDI patients were collected for subsequent genetic comparison purposes, including those of patients with colonization prior and posterior to CDI treatment. To elucidate the genetic relationship, isolates were subjected to Whole Genome Sequencing (WGS) using either the Illumina MiSeq or the HiSeq platform (Illumina Inc., San Diego, CA, USA). After quality trimming, coding core genome regions were compared in a gene-by-gene approach (core genome multilocus sequence typing, cgMLST) with the help of the SeqSphere + software version 7.0.1 (Ridom GmbH, Münster, Germany), using the published *E. faecium* cgMLST target scheme [32]. The clonal relationship of genotypes is displayed via a minimum spanning tree algorithm using the same software and is considered closely related if genotypes differ in three alleles or less. The MLST sequence types (STs) and underlying *van*-genes were extracted from the WGS data in silico.

### 4.6. Statistical Analysis

All data are expressed as absolute numbers or percentage. Categorical data were analyzed using Fischer’s exact test. Statistical significance was assumed at *p* < 0.05.

### 4.7. Ethical Statement

All strategies and investigations were performed in accordance with the national recommendations for surveillance of nosocomial infections and multidrug-resistant bacteria of the German legally-assigned institute for infection control and prevention (Robert-Koch Institute, Berlin, Germany).

## 5. Conclusions

Antibiotic treatment of CDI-affected patients can favor the acquisition of VRE. Therefore, compared to metronidazole, oral vancomycin does not increase the risk of VRE. No specific genetic features underlie the acquisition of VRE. Rather, this results from both antibiotic selection and pathogen transmission, strengthening the essential need of infection prevention bundle strategies consisting of antibiotic stewardship programs and infection control measures.

## Figures and Tables

**Table 1:** Comparison of demographic and clinical characteristics of patients meeting the inclusion criteria and treated with metronidazole or oral vancomycin (*n* = 170).

Characteristic	Value (%)	
	Metronidazole (*n* = 37)	Vancomycin (*n* = 133)
Demographic data		
Median age (years)	58	50
Male gender	22 (59)	72 (54)
Underlying diseases		
Haemato-oncological diseases	22 (59)	57 (43)
Immunosuppressive disease	23 (62)	57 (43)
Hepatic insufficiency	5 (14)	12 (9)
Liver Transplantation	1 (3)	6 (5)
Renal insufficiency	6 (16)	30 (23)
Long term dialysis	1 (3)	12 (9)
Treatment		
Systemic glucocorticoid treatment	6 (16)	36 (27)
Non-CDI-specific antibiotic treatment	20 (54)	79 (59)
Contact to healthcare system		
Average lengths of stay (days)	67	126
Median number of stays	2	4

## Data Availability

Data is contained within the article.
